# Less is more: strategies to remove marker genes from transgenic plants

**DOI:** 10.1186/1472-6750-13-36

**Published:** 2013-04-23

**Authors:** Yuan-Yeu Yau, C Neal Stewart

**Affiliations:** 1 Department of Natural Sciences, Northeastern State University, Broken Arrow, OK 74014, USA; 2 Department of Plant Sciences, University of Tennessee, Knoxville, TN 37996, USA

**Keywords:** Biosafety, Clean-gene technology, Co-transformation, Homologous recombination, Intra-chromosomal recombination, Marker-free, Meganuclease, Negative selection, Site-specific recombination, TAL effector nucleases, Transposons, Zinc finger nuclease

## Abstract

Selectable marker genes (SMGs) and selection agents are useful tools in the production of transgenic plants by selecting transformed cells from a matrix consisting of mostly untransformed cells. Most SMGs express protein products that confer antibiotic- or herbicide resistance traits, and typically reside in the end product of genetically-modified (GM) plants. The presence of these genes in GM plants, and subsequently in food, feed and the environment, are of concern and subject to special government regulation in many countries. The presence of SMGs in GM plants might also, in some cases, result in a metabolic burden for the host plants. Their use also prevents the re-use of the same SMG when a second transformation scheme is needed to be performed on the transgenic host. In recent years, several strategies have been developed to remove SMGs from GM products while retaining the transgenes of interest. This review describes the existing strategies for SMG removal, including the implementation of site specific recombination systems, TALENs and ZFNs. This review discusses the advantages and disadvantages of existing SMG-removal strategies and explores possible future research directions for SMG removal including emerging technologies for increased precision for genome modification.

## Review

Since the first recombinant DNA was successfully created through the removal of a specific gene from a bacterium and inserting it into another bacterium by the use of the restriction enzyme in 1973 [1], recombinant DNA technology has emerged as a powerful tool for editing genes and genetic elements in vectors to produce novel recombinant DNA products. In 1978, scientists from Genentech successfully used engineered bacteria to produce human insulin, which became the first commercial biopharmaceutical product on the market. Following the continuous improvement of DNA technology and the application of *Agrobacterium* for transgene delivery, the first transgenic plants were successfully generated by four independent groups in the early 1980s [2-5]. In 1994, the first commercialized genetically modified (GM) plant product, Calgene’s ‘FLAVR SAVR’ tomato, was sold on the market. Today genetic transformation in plants has become routine; more than 100 agricultural crops have been genetically modified in research stations around the world (http://sbc.ucdavis.edu/B4S/B4S.html). According to the International Service for the Acquisition of Agri-Biotech Applications (ISAAA), the global area used for cultivating transgenic crops has grown to 160 million hectares in 2011, an 94-fold increase from 1996 to 2011. GM technology has not only become an important plant breeding tool, but has great potential humanitarian impact on developing countries for food security [6].

Although transgenic crop adoption has been increasing, the fact that a selectable marker gene (SMG, usually an antibiotic- or herbicide-resistant gene [7]) remains in the genomes of GM plants has raised concerns from both regulatory agencies and the public in certain countries. These concerns include the potential impact on transgenic crops for both food safety and the environment. Among the most controversial concerns is that a SMG coding for an antibiotic resistance originally from a bacterium might be horizontally transferred from GM plants back to bacteria causing new antibiotic resistance problems, for example, in the human- or animal gut environment. The main question is can pathogenic microbes receive GM plant SMGs through horizontal gene transfer and cause new problems, i.e., increasing the possibility of compromising the clinical effectiveness of antibiotic drugs? The increasing number of cases of bacteria that have evolved antibiotic resistance, such as methicillin-resistant *Staphylococcus aureus* (MRSA) (http://www.cdc.gov), has undoubtedly led to consumer concern.

The second concern is gene flow from GM crop to another crop or wild relative, and especially the introgression of SMGs where they might either increase fitness and persistence [8] or render a population at risk; i.e., the worst case scenario, extinction [9]. Although gene flow is a natural process [10], SMGs do not naturally exist in plants and they confer novel traits such as antibiotic- or herbicide-resistance. Therefore, it has been argued that it is desirable to confine transgene(s) in GM crop populations of interest to minimize gene flow to other related species. The gene flow of an herbicide-resistance gene (*bar*) has been studied in Canada in herbicide-resistant canola (*Brassica napus*) fields. Transgene flow from commercial herbicide-resistant canola to its weedy ancestor *Brassica rapa* was observed [11]. Both escaped transgenic *B. napus* and hybrids from hybridization of *B. napus* to its related species were identified outside the crop field boundaries. A more recent study showed that a drift level of herbicide (glyphosate) can also function as a selective agent to increase the persistence of transgenes in environment [12].

Because of these concerns, strict regulations are usually applied in the facilities that work on transgenic organisms. In the case of the USA which has the most GM crops grown, NIH guidelines for biosafety containment levels for plants (BL1-P to BL4-P) must be followed for researchers handling recombinant DNA in plants in laboratories (http://oba.od.nih.gov). In addition, before the release of GM plants from laboratories for field trials, permits are required and strict rules are set out for transgene containment (http://usbiotechreg.nbii.gov). Although, so far, research results have not shown evidence that food from transgenic crops has negative impacts on human or animal health [13], and although transgene-flow can be reduced by careful field designs [14], much uncertainty still exists. Some concern and uncertainty might be assuaged by the removal of SMGs once they are no longer needed. Therefore, the production of SMG-free transgenic crops, a so-called ‘clean-gene technology,’ is becoming an extremely attractive alternative and could be a positive factor to contribute to the public acceptance of transgenic crops.

Other benefits from the removal of SMGs from transgenic crops are as follows. (1) The same SMG can be recycled for reuse in subsequent plant transformation projects; e.g., incremental transgene stacking [15,16]. There are few candidate SMGs-selection agent pairs for most crops [17,18], thus, it would be expedient to recycle SMGs. (2) The removal of metabolic burden from unwanted SMG expression on the plants, especially those that are highly expressed. For example, the SMG FLARE was reported to accumulate to 18 % of total soluble cellular protein in plants whose plastids were transformed [19]. Therefore, the development of strategies to produce SMG-free transgenic plants is an important objective in plant biotechnology research.

In this review, we discuss several approaches that have been developed to generate SMG-free GM plants, with a focus on what we consider to be the most promising recently developed technologies. We discuss site-specific recombination systems, which have been used to remove SMGs from model plants and crops species [20]; commercial SMG-free maize ‘LY038,’ which has increased free lysine in the germ portion of the grain [21]. We also discuss the use of the most recent zinc finger nuclease (ZFN) and transcription activator-like nuclease (TALEN) technologies to remove SMGs, and the possibility of combined use of ZFNs and site-specific recombination to remove a gene from a desired genomic location.

There are three basic approaches in producing SMG-free transgenic plants. They are: (1) avoidance of SMGs, (2) integration of an SMG and genes of interest (GOI) on unlinked genomic loci during transformation so that the SMG can be segregated away from the GOI, and (3) integration of an SMG and GOI on the same locus, but molecular tools are used for SMG removal. We will assess the current progress for each of these strategies and speculate on the future utility of each.

## Avoidance of SMGs in plant transformation

Because the transformed plant cells carry T-DNA(s) with known sequence, it is possible to distinguish transformed from non-transformed plants using PCR. De Vetten *et al.* used two different *Agrobacterium tumefaciens* strains (LBA4404 and AGL0) to transform potato *cv.* Kanico without any selection [22]. The transformation frequency was 0.2%, based on PCR for putative transgenic shoots, using LBA4404 and 4.5% for AGL0. Stable transformation frequency was reduced approximately by half in this system. Genetic transformation of barley without using any SMG has also been performed [23]. The transformation frequency without selection is 0.8% when *Agrobacterium* inoculation of ovules was performed. For comparison, the transformation with selection (hygromycin) frequency was 3.1%. Kim *et al.* transformed potato with chloroplast-targeted *SOD* and *APx* genes, driven by oxidative-stress-inducible promoters [24]. The transformation efficiency was 2.2% without any selection. Recently, Li *et al.* reported a tobacco transformation efficiency of 2.2%-2.8% for the most effective binary vector they used in the experiment without selection and over 90% transformation efficiency with selection [25]. To date, the data demonstrate that although it is achievable to obtain transgenic plants without using selection, the majority of the screened plants would be un-transformed, and thus, not using an SMG at all is very laborious and useful for just a few plant species.

## Co-transformation (transfer GOI and SMG separately) and segregation

### Two T-DNAs on two binary vectors

For this strategy, the gene of interest (GOI, such as a trait gene) and an SMG are cloned into two separate transformation vectors, and subsequently the GOI and the SMG are transferred separately into plant tissues using *Agrobacterium*-mediated transformation [26,27] or particle bombardment [28]. The rationale of this approach is that after transformation, a portion of the resistant transgenic plants surviving antibiotic (or herbicide) selection should have also taken up the GOI-containing T-DNA cassette. Those transgenic plants with both SMG and GOI are allowed to set seeds for the next generation. By segregating away the SMG which is, hopefully, unlinked to the GOI in the subsequent generation, plants with only the GOI can be obtained.

**Figure 1:**
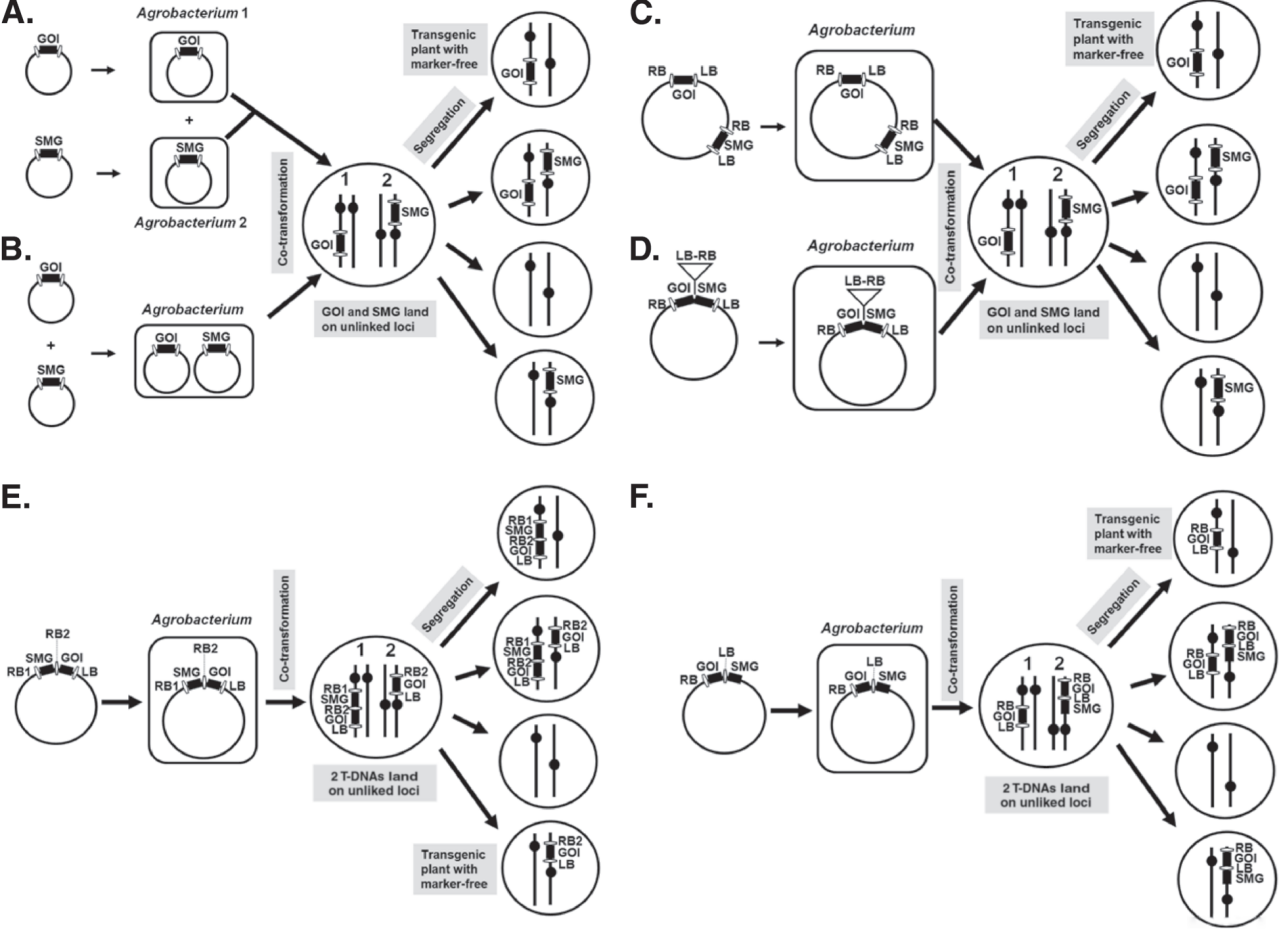
**Approaches for co-transformation to produce selectable marker gene (SMG)-free transgenic plants.** After the GOI and SMG are integrated on unlinked loci, marker-free transgenic plants can be obtained in the sequential generation after genetic segregation. (**A**) Mixture method of *Agrobacterium* co-transformation. Two *Agrobacterium* strains each contain a transformation vector. One vector has the GOI and the other has the SMG, which are used together to co-transform plants. (**B**) One-strain method of *Agrobacterium* co-transformation. One *Agrobacterium* contains two transformation vectors: one with the GOI and the other one with the SMG. (**C**) Super binary vector for co-transformation. The super binary vector contains separated T-DNA constructs: one with the GOI and the other with the SMG. (**D**) Two pairs of right borders and left borders used for co-transformation. A small right border-left border fragment is inserted into the polylinker of a standard binary vector in between a GOI and an SMG, which are already present in another set of left border and right border. (**E**) Double ‘RB’ T-DNA approach for co-transformation. The vector is designed in such a way that two right borders (RB1 and RB2) and one left border (LB) are presented as depicted in the figure. Two T-DNA fragments (RB1-SMG-RB2-GOI-LB and RB2-GOI-LB) may be transferred and be integrated on unlinked loci. (**F**) ‘Read-through’ method for co-transformation. The SMG is placed outside the left border. T-DNA fragment transfers to plant genome could be RB-GOI-LB or RB-GOI-LB-SMG (a ‘read through’ product). If RB-GOI-LB or RB-GOI-LB-SMG is integrated on unlinked loci, marker-free transgenic plants can be obtained through genetic segregation. Abbreviations. RB: T-DNA right border, LB: T-DNA left border, GOI: gene of interest, SMG: selectable marker gene.

*Agrobacterium*-mediated transformation has been used more often than particle bombardment for co-transformation. Two methods to co-transform the separate vectors by *Agrobacterium* have been reported. (1) The *mixture method* uses co-transformation of two different *Agrobacterium* strains each with a transformation vector whereby one carries the SMG and the other the GOI (Figure 1A). (2) The *one-strain method* transforms an *Agrobacterium* strain that carries two expression vectors, whereby one carries the SMG and the other the GOI (Figure 1B) (http://www.isaaa.org. Pocket K. No. 36 Marker-Free GM Plants). De Block and Debrouwer evaluated a large number of canola plants that were co-transformed using *Agrobacterium* and found that when the T-DNAs were delivered by the mixture method, co-transformation occurs often, but in most cases, the T-DNAs were genetically linked [26]. In contrast, Komari *et al.*[27] reported that the co-transformation frequency of two T-DNAs was greater than 47% using the one-strain method. Up to 25% of the co-transformed T-DNAs were unlinked.

### Two T-DNAs on one binary vector

A single *Agrobacterium* strain that harbors dual T-DNAs for transformation on a single plasmid, known as the *two-T-DNA* strategy has also been used with the intention of the two T-DNAs integrating independently as they do for co-transformation in biolistics [28]. Two variants are the super binary vector strategy and the twin T-DNAs strategy.

#### The super binary vector method

In this strategy, two T-DNA constructs are produced on a single binary vector in such a way that they are separated with an intervening DNA fragment (Figure 1C). The GOI and the SMG are transferred into plant cells independently through *Agrobacterium*-mediated transformation. Komari *et al.*[27] developed a 50–55 kb ‘super binary’ transformation vector with two T-DNA regions that were separated by at least 15 kb. More than 50% progeny contained only the GOI, without the SMG.

Streamlined binary vectors, such as the ‘twin T-DNA’ standard binary vector system [29], were created for co-transformation to obtain SMG-free transgenic crops, with the goal of increasing efficiency by decreasing total vector size. Subsequently, Zhou *et al.*[30] produced an intermediate-sized double T-DNA binary vector from two popular binary vectors (pBin19 and pCAMBIA2300) for co-transformation to produce SMG-free transgenic tobacco. Most recently, a standard binary vector (~ 11 kb) containing two independent T-DNA constructs (one with a SMG and one with the GOI) was used for co-transformation and successfully produced SMG-free transgenic sorghum [31].

#### The twin T-DNA method

As mentioned above, the super binary vectors are large and require an unwieldy *in vivo* homologous recombination method to assemble the constructs. Matthews *et al.*[29] developed an improved system called ‘twin T-DNAs’ to improve the shortcoming of ‘super binary vectors.’ In this system, a small so-called ‘right-border/left border’ T-DNA ‘twinning’ insert was inserted into the polylinker of a standard binary vector in between a GOI and a SMG which were already in another set of left border (LB) and right border (RB) (*LB-GOI-polylinker-SMG-RB*). The resulting construct is *LB-GOI-RB/LB-SMG-RB*, with two adjacent T-DNAs without any intervening sequence (Figure 1D). Using these two adjacent T-DNA constructs for co-transformation experiments, a co-transformation frequency of 66% was obtained, and 24% of the progeny segregated in the next generation to yield SMG-free transgenic barley. Taken together, approximately 16% useful independent co-insertion events among transformed lines were produced.

#### One double right-border T-DNA on one vector method

Instead of using multiple T-DNAs, a single T-DNA with a special design on the borders can also be used for co-transformation. To further extend the ‘twin T-DNA’ concept, Lu *et al.* developed a system called the double right-border (DRB) binary vector [32]. The DRB T- DNA construct contains the following components: *RB1-SMG-RB2-GOI-LB*. Two types of T-DNA inserts, one initiated from RB1: ‘*RB1-SMG-RB2-GOI-LB*’ and the other one from RB2: ‘*RB2-GOI-LB’*, were expected to be produced and integrated in the genome. The unlinked inserts can segregate away from each other in the subsequent generation and allow the selection of progeny with only the GOI (Figure 1E).

#### One T-DNA (with SMG outside the borders) on one vector

Another variant, described by Huang *et al.*[28], places the SMG outside the T-DNA borders, on the backbone of the binary vector, leaving only the GOI within the T-DNA. By taking advantage of the fact that the right border enhances the T-strand initiation while the left border enhances the T-strand termination [33], and that there exists the possibility of the left border readthrough, whereby the elongation of T-strand does not stop at the left border but includes the DNA fragment outside the left border [34], two of the inserts, RB-GOI-LB and RB-GOI-LB-SMG are expected to be produced and inserted into the genome of transgenic plants. By segregating away from each other, they successfully generated SMG-free transgenic maize plants (Figure 1F).

### Co-transformation using particle bombardment

The co-bombardment method is analogous to the two-T-DNA *Agrobacterium*-mediated transformation method. Using co-bombardment, both plasmids with the SMG or GOI are coated onto the gold particles for shooting into plant tissue cells. Although unlinked integration of transgenes has been reported resulting in a few SMG-free plants, not many studies have produced marker-free plants using this system; none with high efficiency [35-38]. This is mostly because direct DNA transfer through bombardment frequently results in the insertion of multiple copies and rearrangement of the transgene into a genome or a single locus to form complex inserts [39]. The presence of multiple copies of transgenes, especially those arranged in inverted repeats, not only can induce transgene silencing [40,41] but also complicates the segregation process. Recently, Prakash *et al.* have taken an improved strategy of co-bombardment by using “the linear essential DNA (not the whole plasmid)” and “limited amount of DNA (for bombardment)” approach to produce SMG-free transgenic corn [28]. This minimizes the shortcomings caused by using co-bombardment mentioned above. The linearized DNA cassettes were isolated from plasmids *via* appropriate restriction-enzyme digestions. Particle bombardment was performed with only 2.5 ng of the SMG (*npt*II) cassette DNA and 15 ng of the GOI cassette DNA per shot, amounts which are much lower than the 0.6-1 μg/shot suggested by the manufacturer of the *Bio-Rad* PDS-1000 Biolistic Particle Delivery System. With this approach, 28 SMG-free maize plants were recovered from the progeny of 103 R_0_ plants containing both co-transformed constructs.

### Improved co-transformation strategies for SMG removal

Although using co-transformation for SMG removal is not complicated, PCR screening can be time-intensive and tedious. Several strategies have been implemented to increase co-transformation efficiency. They include (1) the combined use of a negative selection marker with the positive selection marker, (2) the practice of ‘transient’ selection on the positive selection marker and (3) the use of androgenesis coupled with a co-transformation technique.

#### Employment of positive–negative selection with co-transformation system

**Figure 2:**
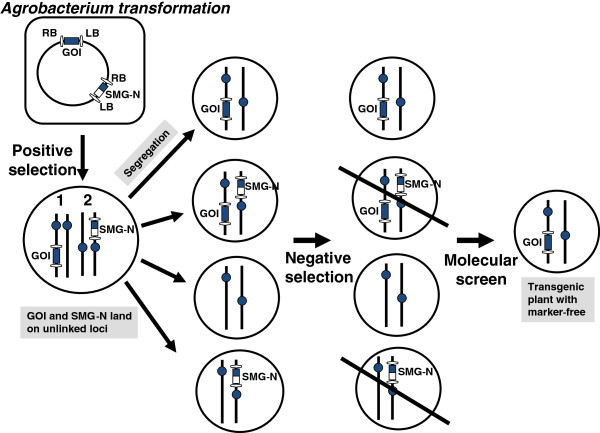
**Positive-negative selection scheme in producing SMG-free transgenic plants.** A negative selectable marker gene (‘N’) is build next to a positive selectable marker gene (SMG), SMG-N, as depicted in the figure. After genetic transformation, explants are first selected with the positive selectable marker. Seeds of next generation are then selected with a negative selectable marker to remove 50% plants which still have SMG-N. PCR is then followed to screen marker-free transgenic plants. Abbreviations. RB: T-DNA right border, LB: T-DNA left border, GOI: gene of interest.

In a co-transformation experiment, a positive SMG (such as *nptII* or *bar* gene) is used to co-transform with a GOI. The selected T_0_ plants (either with SMG-only or with both the SMG and GOI) are then allowed to set T_1_ seeds to allow segregation to select the GOI-only transgenic plants. However, without the assistance of negative selection markers this practice requires a PCR-based screen on a large scale to isolate small portions of SMG-free/GOI-only transgenic plants from the segregating population [42]. Negative selection markers are used to optimize transformation efficiency; opposite to positive SMGs, they kill the transformed cells. This positive–negative selection method usually places a negative SMG next to a positive SMG in the construct [42-47]. The positive SMG is used for selection of the T_0_ transgenic plants, and the negative SMG is then used to remove plants still harboring the ‘negative SMG’ cassette from the subsequent T_1_ segregation population, and greatly reduce the transgenic plants for researchers to screen for the GOI-only transgenic plants (Figure 2).

The most widely used negative SMG for transgenic plant selection is the *codA* gene from *E. coli*[42-46,48-50]. The *codA* gene encodes *cytosine deaminase,* which converts the non-toxic 5-fluorocytosine (5-FC) into the toxic 5-fluorouracil (5-FU). Development of *codA*-expressing seedlings is strongly inhibited by germinating the seeds in the presence of 5-FC. Other negative SMGs such as *iaaH*, *argE* and cytochrome *P450*_*SUI*_ are also used to kill or inhibit the growth of the transgenic plants. The indole acetamide hydrolase (*iaaH*) gene product converts naphthalene acetamide (NAM) to naphthaleneacetic acid (NAA), resulting in the inhibition of normal plant growth [51,52]. The *Escherichia coli* ornithine deacetylase gene *argE* is an acetyl transferase that deacetylates inactive N-acetyl phosphinothricin (N-acetyl-PPT), a chemical that is not toxic to plants, and produces phosphinothricin (PPT), the active ingredient of the Basta® non-selective herbicide [53,54]. Bacterial cytochrome *P450*_*SUI*_ converts non-toxic pro-herbicide R4702 into cyto-toxic herbicide R4702 [50,55,56]. Most recently, a transcriptional activator protein gene (*TrAP*) from *Mungbean yellow mosaic virus* (MYMV) was also used as a negative SMG in a positive–negative selection system to generate SMG-free tobacco plants [47].

#### Transient positive selection of co-transformed plants

Most marker-removing strategies in co-transformation system require two steps: first, the GOI construct and SMG construct are introduced into cells to obtain T_0_ transgenic plants with proper positive selection; then the SMG is removed by genetic segregation in the T_1_ generation. If GOI and SMG could be separated early in the T_0_ generation, there would be a two-fold advantage: (1) faster breeding time, (2) obviating the segregation process to remove the SMG in the T_1_ generation. This would be of special benefit for asexual-propagated plants that do not go through segregation. Transgenes transferred into plant cells are not necessarily stably integrated into the genome. In rare cases, an un-integrated T-DNA (with SMG) and a stably integrated GOI may co-exist in some cells. With a short selection phase, those cells may survive on the select medium with the transient expression of the SMG. While performing co-transformation using a positive–negative marker selection, Ramana Rao and Veluthambi [47] recovered 4.4% (5/114 plants) of GOI-only, SMG-free, transgenic plants in the T_0_ generation with a ‘*transient*’ (2–4 weeks) period of positive selection. Dutt *et al.* used a transient positive-selection method to obtain SMG-free, GOI-only, transgenic grapevine from T_0_ generation [46].

#### Androgenic segregation after co-transformation for marker free plants

In androgenesis pollen development is arrested to force them towards a somatic developmental pathway. *In vitro* androgenesis can be achieved using microspores leading to the formation of haploid cells and plants either by direct embryogenesis or *via* callus formation [57]. The SMG-free, GOI-only transgenic plants can be obtained through androgenic segregation by anther culture after co-transformation. Subsequent to co-transformation with unlinked T-DNAs, anthers from the regenerated transgenic plants can be used for induction of androgenesis through anther culture. Since some haploid chromosomes can spontaneously divide and diploidize during anther culture, this practice could be used to produce genetically true-breeding, homozygous doubled-haploid (DH) and marker-free plants [58]. Li and his co-workers [59] have used the co-transformation and anther culture approach to rapidly generate SMG-free doubled-haploid transgenic rice in one year with an efficiency of 12.2%.

### Transposon-based SMG removal

Transposons are comprised by genetic elements that can “jump” around in the genome of an organism. The best characterized transposons are those of the *Ac/Ds* family, which were first discovered in maize by Barbara McClintock when studying phenotypic markers of maize germplasm [60]. *Ac* and *Ds* are two related elements. *Ac*, short for *activator,* encodes the enzyme transposase, while the *Ds*, short for *dissociation,* is a deletion version of *Ac* element. Both elements share 11-bp terminal inverted repeat sequences (TIRs). Since the *Ac* element can produce a 102-kD functional transposase to move itself around in the genome; it is termed *autonomous*. On the other hand, the *Ds* element requires *Ac* to produce transposase for transposition; therefore, considered *non-autonomous*. Approximately 200 bp on each end of the element is necessary for transposition. Transposase binds to the hexamer motif AAACGG within these 200 base pairs. Excision of *Ac/Ds* elements leaves a characteristic footprint (minor sequence changes) at the donor site.

**Figure 3:**
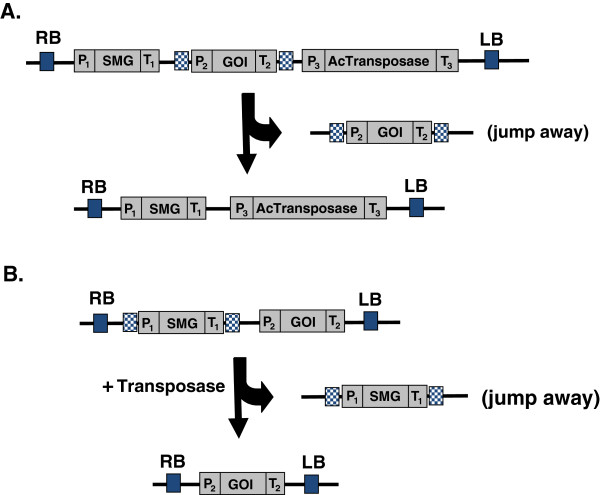
**Strategy for using Ac transposon element to produce SMG-free transgenic plants.** (**A**) T-DNA contains both the GOI (flanked by transposase-recognizing sites) and the transposase is transformed into plants. When the transposase is activated, the GOI cassette will be excised and insert into a different locus. If the GOI cassette lands at an unlinked locus, the SMG-free transgenic plants can be obtained after segregation in the subsequent generation. (**B**) T-DNA containing both the SMG (flanked by transposase-recognizing inverted repeat sequences) and the GOI is transformed into plants. When transposase activity is provided, the SMG cassette can move away and insert into a different locus. If the SMG and GOI cassettes land at an unlinked locus, the SMG-free transgenic plants can be obtained after genetic segregation in the sequential generation. Abbreviations. RB: T-DNA right border, LB: T-DNA left border, GOI: gene of interest, SMG: selectable marker gene. P_1_, P_2_ and P_3_: promoters, T_1_, T_2_ and T_3_: terminators.

The *Ac* element has the ability to move to new locations within a genome, so the GOI or the SMG can be placed within the ‘jumping’ sequence and eventually being excised and re-insert into other locations in the genome. An example of a vector designed for this purpose is described in Figure 3A. The GOI cassette flanked by two terminal-inverted repeats of the maize *Ac/Ds* transposon system was build next to an SMG and *Ac* transposase cassette. After the T-DNA is transferred into plants, the *Ac/Ds* system becomes active. The encoded transposase recognizes the inverted repeat signals at both ends of the GOI and catalyze the relocation of the GOI in the genome. Once the GOI jumps away from the SMG in the genome and becomes genetically unlinked, segregation can result in GOI-only plants. Cotsaftis *et al.*[61] have used this approach to obtain insect-resistance rice without an SMG. It is worth noting that the additional advantage of using a GOI re-position-mediated-by-transposon strategy is that a large population of transgenic lines with the target gene (GOI) can be generated for evaluation for position effects, which is very useful for species which are not amenable for efficient genetic transformation, such as wheat.

The SMG could also be placed in between the inverted repeat signals, instead of GOI (Figure 3B). It was found that about 10% of the *Ac* elements that are excised do not re-insert and therefore disappear from the genome or re-insert into a sister chromatid that is subsequently lost during somatic segregation. Ebinuma *et al.*[62] employed the *Ac* element to remove the isopentenyl transferase gene (*ipt*), which was used for visually distinguishing transgenic plants from non-transgenic plants by identifying the *ipt* “shooty” phenotype. In this study, a T-DNA was constructed to contain an *Ac* element linked to an SMG cassette (35S promoter-*ipt* gene-terminator) and a GOI cassette. When the *Ac* element transposed, the *ipt* gene transposed too and was subsequently removed, which resulted in a T-DNA that contained only the GOI cassette. Six months after infection, the researchers had obtained SMG-free transgenic tobacco plants – from which *Ac* had disappeared from 4.8% of the transgenic lines. If an excised element does not re-insert and simply disappears from the genome, the genetic segregation process is not needed. This would be a useful approach to produce asexually propagated plant species containing no SMG. More recently, an elegant study combined the *piggyBac* transposon system and the ϕC31 site-specific recombination system to produce SMG-free transgenic lines and stabilized (demobilized) an unstable transgenic gene cassette by eliminating its 5’end transposon TIR in fruit fly [63]. This approach can be useful for transposon-mutated plant lines.

### Site-specific recombination-mediated SMG removal

Site-specific recombination systems have advanced in diversity and applications in recent years. Applications include SMG removal. Site-specific recombination systems are common in prokaryotes and lower eukaryotes such as yeast and serve various biological functions [64]. The recombinase protein catalyzes recombination of DNA between two recognition sites. The outcome of the recombination can be site-specific excision, integration, inversion or translocation depending on the position and the relative orientation of the two recognition sites on the DNA molecules (either linear or circular form), and the type of reaction is dependent on enzyme type. Cre-*lox* site-specific recombination system was the first to be used for SMG excision in tobacco [65,66]. Since then, many labs have successfully used Cre-*lox* or other later-identified site-specific recombination systems for SMG removal in plants (reviewed by Gidoni *et al.*[67]).

SMG removal using recombinase systems have proven effective. A common feature in these studies is the production of transgenic plants with a SMG flanked by two recombination sites oriented in the same direction. Upon expression of the corresponding recombinase, a site-specific recombination event excised the SMG residing between the recombination sites.

#### Constitutive expression of a recombinase gene using plant hybridization or retransformation

**Figure 4:**
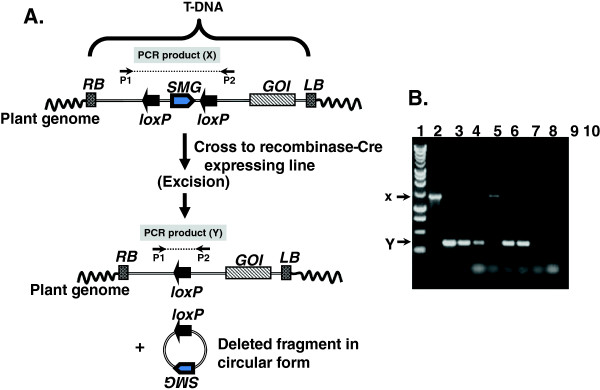
**PCR screening of Cre-*lox*-mediated SMG excision events in plant genome.** (**A**) Using primers P1 and P2 designed from outside the two *loxP* sites, PCR product ‘X’ (parental band) was amplified when no excision occur, while ‘Y’ band is produced when excision events occur. (**B**) Lane 1: DNA markers, lane 2: parental band (X), lane 3: positive control band (Y) (obtained from transient assay in bacterial cells), lanes 4–9: excision event detection, lane 10: no DNA. Abbreviations. RB: T-DNA right border, LB: T-DNA left border, GOI: gene of interest, SMG: selectable marker gene.

Initial studies using site-specific recombination systems to remove SMGs in plants used either a hybridization strategy, or a re-transformation strategy. For the hybridization strategy, the target plant is produced with a GOI and the SMG flanked by recognition sites (Figure 4A). The recombinase-expressing transgenic plant is hybridized with the target plant so that hybrid F_1_ plants will have SMGs excised. F_1_ plants containing both transgenes are used to screen for deletion events (in this case, SMG removal) (Figure 4B). Transgenic plants with recombination events are then backcrossed to wild type for obtaining offspring with germ line transmission of the final product of excision and absence of the recombinase gene. For the re-transformation strategy, after target line plants are produced, they are re-transformed with a recombinase-expressing cassette (for which a different SMG is needed). The re-transformed lines are screened for the presence of the recombinase-expressing gene. Transgenic lines with the gene are allowed to set seeds, and plants derived from these seeds are tested for germ line transmission of the SMG-excised, final transgenic product. The recombinase gene can then be removed from the SMG-free GOI lines by genetic segregation if the recombinase gene and GOI are not linked. Although marker-free transgenic crop plants have been produced using these two processes, there are disadvantages to each. The hybridization strategy is restricted to sexually propagated species. For trees, long generation times make crossing schemes impractical. On the other hand, the re-transformation method is lengthy and requires twice the exposure to tissue culture-hormonal conditions, which could increase mutagen risk. There are also reports that overexpression of the recombinase gene might cause abnormalities in the transgenic plants [68], but this could be a risk for all recombinase-based systems. To avoid recombinase-mediated off-effects, the recombinase could be expressed transiently, inducibly, developmentally, and it could also be engineered for auto-excision.

#### Transient expression of the recombinase gene

To deliver recombinase genes into plant cells for transient, high-level expression of recombinase proteins, without integration of the recombinase genes into the genome, plant virus vectors [69,70] and *Agrobacterium* T-DNA vectors [71] can serve as vehicles. Kopertekh *et al.* reported using a Potato Virus X (PVX)-based vector to transiently express the Cre recombinase gene to remove a SMG from transgenic *Nicotiana benthamiana*[69]. Kopertekh and Schiemann also reported using an agroinfiltration technique to perform Cre-mediated SMG removal in *N. benthamiana*[71]*.* The efficiency of obtaining the regenerants with the *bar* gene excised was 34%. Among these 20% of the plants were caused by the expression of a stably integrated *cre* gene, whereas the remainder (14%) resulted from transient *cre* gene expression. Particle bombardment has also been used. Srivastava *et al.* co-bombarded a *cre* gene construct and an integration vector into maize callus containing the a target construct for site-specific integration [72]. They found that 60% of the single-copy-transgene-inserted plants lacked the recombinase gene, indicating that *cre* gene expression was transient.

#### Induced expression of a recombinase gene

**Figure 5:**
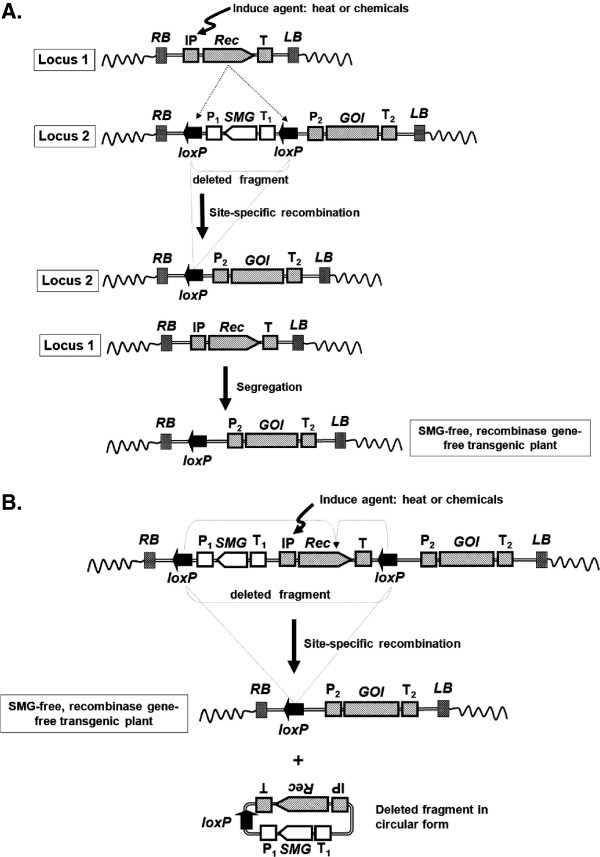
**Strategy of using chemicals or heat to induce SMG-removal in transgenic plants.** (**A**) The T-DNA contains an inducible promoter (IP) and a *cre* recombinase gene (Rec) that is transformed into a plant and reside at locus 1, while the second T-DNA cassette containing the SMG and GOI is transformed and integrates on locus 2. When the induce agent (heat or chemicals) is applied, Cre recombinase protein is produced and excises at the two *loxP* sites, which removes the P_1_-SMG-T_1_ fragment. If locus 1 and locus 2 are unlinked, SMG-free transgenic plants can be obtained after segregation in the subsequent generation. (**B**) Autoexcision scheme. In the autoexcision strategy, the SMG and the *cre* recombinase gene [which uses an inducible promoter (IP)] are constructed within two *loxP* sites. Once the inducible promoter is activated, the whole unit within the two *loxP* sites is removed altogether. Abbreviations. RB: T-DNA right border, LB: T-DNA left border, GOI: gene of interest, SMG: selectable marker gene, IP: inducible promoter, P_1_ and P_2_: promoters, T, T_1_ and T_2_: terminators.

In order to further control the excision process to remove the SMG at a certain time and avoid constant expression of recombinase genes, recombinase genes have been placed under the control of inducible promoters. These include heat shock inducible promoters [73] and chemical inducible promoters. By activating the promoters with inducers (heat or chemicals), the expression of the recombinase gene can be more tightly controlled, even though existing promoters used for this purpose typically lack regulatory precision. In one strategy, the SMG, the recombinase gene, and the GOI are cloned into a single construct. The recombinase gene under the control of an inducible promoter and the SMG are placed into a cassette flanked by two recombination sites oriented in the same direction, whereas the GOI is placed outside the region flanked by the recognition sites. After transformation and molecular analysis, the transgenic plants are treated with inducers for removal of SMG and recombinase (Figure 5). This strategy of using a heat shock inducible promoter to control timely expression of the recombinase gene has been applied to *Arabidopsis*[74], maize [75], tobacco [76], potato [77] and aspen [78] for site-specific recombination events.

A chemical-induced promoter was used for a recombinase-based SMG removal in *Arabidopsis* by Zuo *et al.*[79], wherein an estrogen-receptor-based transactivator XVE, for the bacterial repressor LexA (X), the acidic transactivating domain of VP16 (V), and the regulatory region of the human estrogen receptor (E) [80] and the Cre-*lox* system were used in combination. In transgenic plants, β-estradiol-activated XVE stimulated expression of the Cre protein under the control of the target promoter; this resulted in Cre-mediated site-specific recombination events and deletion of the unnecessary DNA. The XVE system was also used in aspen with FLP-*FRT* system [81], in rice with Cre-*lox* system [82], in tomato with Cre-*lox* system to produce SMG-free transgenic plants [83,84]. In another system, Woo *et al.* reported auto-excision of SMG from transgenic tobacco *via* a stress-inducible FLP-*FRT* site-specific recombination system [85]. In this system, an oxidative-stress-inducible promoter, a peroxidase (POD) promoter, was fused to the recombinase gene *flp*. Hydrogen peroxide (H_2_O_2_) was used to induce the recombinase gene, and the successful excision of SMGs *via* hydrogen peroxide (H_2_O_2_)-regulated site-specific recombination was observed.

Because heat-shock treatments and chemicals are required for recombinase activation for SMG deletion, these treatments may be limited to certain plant species and might complicate the transformation process [86]. Other problems associated with these inducible systems include low promoter induction, which can lead to incomplete transgene excision, and/or off-effects from leaky expression and unintended excision. Leakiness in promoter activity is common in transgenic plants and has been reported by several laboratories that used chemical- or heat shock-inducible promoters for recombinase gene expression [67]. Therefore, better promoters are needed to improve precision. In the absence of improved promoters a ‘double-lock’ method was employed by Joubès *et al.*[87] to tightly control a heat-shock-inducible promoter (*HSP18.2*) and thus avoid premature activation of the inducible system. This strategy combines the use of Cre-*lox* site-specific recombination system and a mammalian glucocorticoid receptor (GR) for the tightly control on the transcription and translation levels. No deletion was detected when only chemical induction or only heat treatment was used, confirming the effectiveness of the double-lock approach.

Employing a strategy of using the constitutive promoter 35S instead of inducible promoters to drive a recombinase for auto-excision of the SMG along with the recombinase gene at the same time has also been reported. Kondrák and colleagues [88] designed a transformation vector with an auto-excision cassette and one RB of T-DNA to successfully produce SMG and plasmid-backbone-free transgenic plants. However, since 35S is a constitutive promoter, an intron was added inside the recombinase gene so that the recombinase gene would be inactive in *E. coli* or *Agrobacterium* cells, while providing recombinase activity when the intron was removed naturally in plant cells [89].

#### Developmentally-programmed recombinase gene expression

Although inducible promoters offer control over the timing of recombinase expression, tissue-specific promoters can be used to control excision at key development stages. Successful use of pollen-specific or other tissue-specific promoters to drive recombinase gene expression for SMG removal has been accomplished [86,90-94]. Mlynárová *et al.* used the tobacco microspore-specific NTM19 promoter to control *cre* expression for auto-excision in pollen [90]. The system was highly efficient with a failure rate of only 2 out of the 16,800 seeds (0.024%), which were derived from the SMG-free tobacco pollen. Additionally, expression of the *cre* gene was tightly regulated; leakiness of the promoter was not observed. Verweire *et al.* also successfully demonstrated the use of *Arabidopsis* germline-specific promoters from the *APETALA1* and *SOLO DANCERS* genes to achieve genetically programmed, Cre-recombinase-mediated auto-excision for SMG-free plants [92]. More recently, Frédéric *et al.* evaluated seven different germline-related promoters for their suitability in regulating Cre expression in transgenic *Arabidopsis*[93]*.* Five out of the seven promoters, which varied in developmental stages and tissues were able to drive efficient Cre-mediated gene excision. The data also showed that use of these promoters resulted in lower variation in recombination frequency than previously reported for the 35S promoter. These newly tested tissue-specific promoters provide an additional tool for the developmentally-programmed site-specific recombination-mediated SMG removal.

#### Use of site-specific recombination for SMG removal in plastid transformation

Genetic transformation of plastids has become an attractive alternative to nuclear gene transformation when very high recombinant protein levels (may represent up to 70% of leaf protein) and lack of transgene transmission *via* pollen (in many species) are desired. One drawback is that routine plastid transformation procedures in many major crops remains a challenge [95]. Site-specific recombination systems including Cre-*lox* and ϕC31-*att* were used to remove an SMG inserted into plastid genomes [96-98]. The approach generally takes two steps. First, the transplastomic plant target lines with *loxP*-SMG-*loxP* or *attP*-SMG-*attB* construct is transformed into the plastid genomes using particle bombardment. Then the plastid-targeted recombinase gene engineered for expression in the nucleus is genetically transformed through *Agrobacterium* into the transplastomic plants. Expressed CRE or ϕC31 recombinase proteins are then imported into chloroplasts where it excises the plastid-integrated SMG [99]. An alternate strategy used transient expression of the *cre* gene in transplastomic leaves by agroinfiltration, followed by plant regeneration. Approximately 10% of the regenerated plastid marker-free plants did not have the *cre* gene integrated in the nuclear genome [98].

### Use of meganucleases for SMG removal

Meganucleases are homing endonucleases, a large family of DNA nucleases found in eukaryotes, bacteria and archae-bacteria [100]. Homing endonucleases typically are encoded by introns and inteins [101]. Similar to type II restriction enzymes, they cleave double-strand DNA. However, while type II restriction enzymes recognize short nucleotide sequences (3–8 bp), homing endonuclease recognize large target sequences (12–40 bp), which are long enough to occur randomly only with a very low probability. For example, there is approximately one I-SceI cutting site every 7×10^10^ bp [102,103]. The low probability of the presence of endogenous restriction sites decreases the risk of genome fractionation by the meganuclease upon expression.

**Figure 6:**
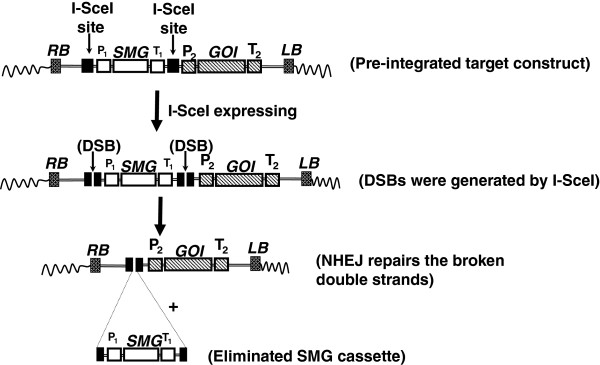
**Meganuclease systems for SMG removal.** Two I-SceI sites were designed to flank P_1_-SMG-T_1_ cassette. When I-SceI expresses, double-strand breaks (DSBs) are generated at both I-SceI sites and release the P_1_-SMG-T_1_ fragment. The broken ends are repaired through NHEJ repairing pathway. Abbreviations. RB: T-DNA right border, LB: T-DNA left border, GOI: gene of interest, SMG: selectable marker gene, P_1_ and P_2_: promoters, T_1_ and T_2_: terminators.

I-SceI is one of the common-used homing endonucleases in research, encoded by the mobile group I intron of the large rRNA gene of *Saccharomyces cerevisiae*[104], which recognize and cleave a recognition sequence of 18 bases (TAGGGATAACAGGGTAAT). Early studies of the meganuclease I-SceI have illustrated how the cleavage activity of this protein initiates homologous recombination events in living cells and demonstrated the recombinogenic properties of chromosomal *double-strand breaks* or DSBs [103], and is now often used to induce gene targeting by promoting homologous recombination through creating site-specific cleavage, a DSB [105]. In mammalian and plant cells, this approach has enhanced gene-targeting frequency by several orders of magnitude [106,107]. In addition to use in gene targeting, meganuclease engineering was also reported for transgene deletion. By using two I-SceI cutting sites flanking a SMG in a transgene construct, it is possible to induce double-strand breaks on both restriction sites to release the SMG upon the expression of I-SceI. The cut ends were re-joined through the *non-homologous end-jointing* (NHEJ) repair pathway, the predominant DSB repair pathway in plants (Figure 6) [108,109].

### SMG removal *via* intrachromosomal homologous recombination (ICR)

The frequency of homologous recombination in flowering plants is very low. For example, according to a previous study, using ICR between two homologous sequences to induce transgene deletion in plants was investigated, and the ICR between closely linked repeated sequences occurs only at a frequency of approximately 10^-6^- 10^-7^[110]. Various approaches have been applied to increase the frequency of homologous recombination for use in gene deletion or gene targeting studies. For example, Bertrand *et al.*[111] reported the spontaneous ICR frequency increased 5- to 20-fold from the original 1.2- 1.5 x 10^-6^ in mammalian cells expressing a mutant _*P*_53 protein. In another example, the introduction of DSB by endonucleolytic cleavage (I-SceI) was also reported to increase the ICR frequency [107,112]. However, when Peter Meyer’s group devised an ICR strategy, which was based on the recombination of the *attP* region of bacteriophage λ, but which did not require the presence of recombinase and other helper proteins, interestingly, high ICR frequency in transgenic plants was observed [113]. In this study, a construct was built to contain two 352-bp λ phage attachment sites (or *attP*) as homologous repeats flanking a SMG. The construct was transformed into tobacco plants, and higher rates of ICR-induced SMG deletion were detected in different parts of two of eleven lines. There were no detectable deletion events in the other nine lines. They attributed this result to a transgene position effect. However, in parallel, the researchers observed that ICR is not always associated with precise homologous recombination between the two *attP* regions; larger deletions (in which regions outside the *attP* sequences were lost, as well) were also detected among the deletion events in the two lines. Illegitimate recombination was proposed as the underlying mechanism for the majority of the deletion events. The high frequency was proposed as that the T-DNAs were integrated into genomic DSBs and stimulate homologous recombination reaction [114], and the only known way to increase homologous recombination by several orders of magnitude– and simultaneously, illegitimate recombination events–is the presence of DSBs [115]. T-DNA can opportunistically exploit genome DSBs as suggested by Salomon and Puchta [116] and Chilton and Que [117].

### Utilization of zinc finger nuclease (ZFN) technology for SMG removal

ZFNs, originally referred to as hybrid restriction enzymes, are fusions of zinc-finger-based DNA recognition modules to endonuclease domains from restriction enzymes [118]. These enzymes are engineered to contain a zinc finger DNA-binding domain (composed, typically, of 3–6 zinc fingers) and the nonsequence-specific DNA-cleavage domain from the *Fok*I type II restriction endonuclease (Fn domain) [115]. The cleavage domain of ZFNs must dimerize in order to cut DNA; therefore, efficient cleavage requires two zinc-finger-binding sites be located in close proximity to one another. Because the zinc finger domain can be engineered to recognize a wide range of novel DNA sequences, a specific ZFN can be designed, produced, introduced and directed to a specific genomic locus for cleavage.

**Figure 7:**
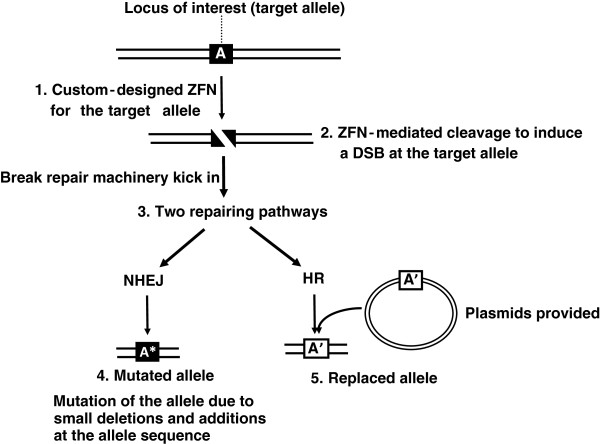
**ZFN technology to modify an allele at a target locus.** 1. Custom-designed ZFN is used to target the target allele ‘A’. 2. ZFN-mediated cleavage to induce a DSB at allele ‘A’. 3. The broken ends were repaired through endogenous repairing pathway. 4. If the broken ends are repaired through NHEJ pathway, a mutated allele (‘A*’) will be generated due to deletions or additions. 5. If HR repairing pathway is used and plasmids with a ‘replacement’ allele (A’, which has partial homologous sequences to allele A) are provided, the allele at the target locus can be replaced to A’.

ZFN technology has been widely used to introduce DSBs at endogenous loci in animal systems to generate allele mutations (a small deletion or insertion of bases at the break sites) and to achieve allele replacement to fix a defective allele, for example, through the NHEJ and homologous recombination repairing pathway, respectively (Figure 7). ZFNs were also deployed for gene excision in animal and plant cells. ZFNs can be used similar to meganuclease-mediated transgene deletion whereby, two concurrent DSBs introduced by ZFNs should be repaired such that the broken ends re-ligated by NHEJ, and the intervening chromosomal segment is excised (Table 1). Two recent reports have demonstrated the use of ZFNs to successfully eliminate DNA fragment in mammalian cells. Two ZFNs were used to target and cleave two different target sites and successfully eliminate the intervening dihydrofolate reductase gene and a 15-megabase chromosomal fragment from the Chinese hamster ovary cells and the human cells, respectively [119,120].

**Table 1:** Summary of key features of DNA-modifying technologies that have been employed for removal of selectable marker genes from plants

**System Name**	**Meganuclease**	**Site-specific recombinase**	**ZFN**
Enzyme involved	Homing nucleases	Site-specific recombinases	Zinc-finger fused Fokl nuclease
System examples	l-Scel	Cre-*lox*, phiC31-*att*	Custom-designed ZFNs
Recognition sites	Specific DNA sequence	Specific DNA sequence	Any endorgenous targeted DNA sequence
Pre-inserted target sites	Needed [random insertion]	Needed [random insertion]	Not needed [locus specific]
Reaction mode	Induce DSB and repair mechanism	Site-specific recombination	Induce DSB and repair mechanism
Product	Not-conserved [through NHEJ repair]	Conserved	Not-conserved [through NHEJ repair]
Concerns of using this technology	Natural existing sites in genomes	Pseudo sites in genomes	Low affinity of ZFN to target DNA
Negative impacts from the concerns above	Genome fractionation	Chromosome rearrangement or deletion	Non-specific DSB induction at the non-specific sites

Compared to applications in mammalian research, ZFNs have been used less-frequently for plant genome editing [121]. In plants, although the technology primarily focuses on introducing local genomic modification [122-126], ZFN technology was recently reported to use for plant SMG deletion. Petolino *et al.* demonstrated that a pre-integrated cassette containing GUS reporter gene flanked by two ZFN cleavage sites was deleted from a stably transformed plant by crossing it with a second plant expressing a corresponding ZFN gene [127]. The same scheme can also be used for SMG removal [127]. Moon *et al.*[94] also proposed this same ZFN method for SMG deletion in plant pollen.

### The limitations and future prospects of existing methods for SMG removal in transgenic plants

Techniques to produce SMG-free transgenic plants have continued to undergo innovation, which is an indicator of the industry need. Another factor driving innovation is that each method we have discussed has limitations.

Using no selection is impractical in most species. There are too many escapes and latent *Agrobacterium* contamination that can confound the results obtained from the system which relies heavily on PCR selection [128]. In addition, no selection can increase the number of chimeric transgenic plants recovered [25]. Therefore, simply not using SMGs for most agriculturally-important plant species will probably not be commercially viable.

At the other end of the spectrum, the co-transformation approach is probably the simplest method to obtain SMG-free transgenic plants. Many isolates of *A. tumefaciens* contain more than one T-DNA, and crown gall tumors often contain multiple T-DNAs [129]. Also, it is generally less tedious to construct a vector with only a GOI or SMG in each T-DNA. However, there are disadvantages to this approach. Success is tied with having an efficient transformation system, which is typically not the case for most elite crop cultivars. Also, if the two constructs are integrated into the same locus or closely linked loci, it could be nearly impossible to segregate them away from each other. This is especially true of the ‘twin T-DNA’ strategy where the two T-DNAs are physically linked to each other. Third, even with a relatively efficient co-transformation system, many putative transgenic plants must be screened, although this drawback can be addressed by the combined use of positive–negative selection systems. Finally, co-transformation-based SMG removal is typically applied on sexually propagated plants whose genomes undergo recombination and segregation, not to vegetatively propagated plants. However, a recent approach using transient positive selection coupled with long negative selection recovered a few SMG-free GOI-only transgenic plants at the T_0_ generation for vegetatively propagated plants, but it is not an efficient system [47].

A transposon-based approach to remove SMG away from GOI or remove GOI away from SMG is an attractive method, but it also has some drawbacks. The first is a transposon system must exist in the laboratory for the species of interest. In addition, transposon systems typically are not very precise and can take a long time for the repeated insertion-and-excision cycles to delete the SMG; the process itself can lead to mutations and increase the genomic instability in transgenic plants [130].

Although the meganuclease-based and ICR-based methods to eliminate SMG are achievable *in planta*, they have not yet been widely explored as other the methods above. The frequency of occurrence of meganuclease rare-cutting sites in crop genomes is one concern from a non-target mutation perspective. Another concern is the induction of various sizes of deletions at the DSB location from DSB-induced NHEJ repairing process may truncate neighboring genes [116]. Using the ICR approach to remove SMG seems relatively simple, as no expression of a heterologous recombinase is required. It is especially attractive for vegetatively propagated crops because no segregation is required. However, because the mechanism of excision of SMG between the two *attP* sequences (bacteriophage λ *attP* site in the published case) is not fully understood, the activity of using *attP* sequences as recombination substrates needs to be demonstrated in crop species. In addition, it would be interesting to explore if other *attP*-like sites from other site-specific recombination systems (such as in Bxb1-*att*) or the other bacteriophage λ attachment site *attB* can be used in this application.

An SMG can be eliminated from pre-integrating a *GOI-ZFN cutting site-SMG-ZFN cutting site* cassette upon the expression of a custom-designed ZFN. However the complicated design and intensive testing of the ZFN arrays might limit the scope of usage of this technology. In addition, the intellectual property rights for plant applications rest in one company. A better-studied site-specific recombination system could be used for the same purpose. Nevertheless, since ZFNs can be designed to bind and cleave a wide range of endogenous DNA sequences, it will be a powerful tool to generate gene mutation and endogenous chromosomal fragment deletion and might find other broad applications in basic research and biotechnology soon in the future [131].

The various site-specific recombination approaches to remove SMGs is most likely to be widely applicable. Numerous labs have successfully employed site-specific recombination to produce SMG-free transgenic plants including major crops [67]. The first Cre-*lox* system-mediated SMG-free commercial corn LY038 was also produced and approved for marketing [132]. Using site-specific recombination systems to remove SMGs seems to be a promising approach. However, to broadly use this technology in agriculture in the future, some issues need to be addressed and further improvements of these systems may be needed.

#### Recombinase-caused plant cell toxicity

Recombinase expression can cause abnormal phenotypes in plants [68]. Similarly, undesired effects following the overexpression of site-specific recombinases in mammalian cells have been documented [133]. Naiche *et al.*[134] observed that Cre activity causes widespread apoptosis and lethal anemia during embryonic development, and Liu *et al.*[135] found that ϕC31 integrase induces a DNA damage response and chromosomal rearrangements in human adult fibroblasts. To avoid over-expression of the recombinases, transient approaches to skip stable integration of recombinase genes could be one way around the problem.

#### The fate of the excised SMG cassette

The general consensus is that the excised SMG fragment, usually in circular form, will naturally be degraded in the cell. However, Srivastava *et al.*[136] reported that rare cases (4 out of 72 plants) of excised SMG fragment remains in the cells as an extra-chromosomal circular molecules for several generations in wheat. In this case, the excised SMG fragment which contained a *bar* resistant gene (they referred to it as the ‘*bar circle’*) was detected with Southern blot analysis even in the progeny of F_3_ and F_4_ generations without the presence of *Cre* gene. Furthermore, from their study, the *bar circles* might have undergone replication, thus these events would need to be screened against so that SMG fragments are not inherited to progeny and in food. A site-specific recombination system for plastid genetic transformation to remove a SMG [137] might be compromised if excised DNA circles could remain in the plastid genome, which can occur up to 10,000 copies per cell [138].

#### The endogenous pseudo-sites and genome instability

Cryptic recombination-sites or pseudo-sites are native DNA sequences that are partially identical to the sequence of native site-specific recombination sites and can serve as substrates for the activity of recombinases and cause unintended recombination. Cryptic sites were reported present in yeast, mammalian and plant genomes. In yeast studies, recombination with pseudo-sites occurred at a very low frequency [139]. However, conducted in the *E. coli* system, Calos’s group discovered that the cloned pseudo-*loxP* sequences from the human or mouse genome could support Cre-mediated recombination at up to 100% of the efficiency of the native *loxP* site [140]. A human Factor IX (hFIX) gene was also reported permanently integrated into two pseudo-*attP* sites, whose sequence is partial identical to the native *attP* sequence, in the mouse genome mediated by ϕC31 site-specific recombination system [141]. Pseudo-*attB* sites for site-specific recombination system A118 were also reported present in the human genome [142]. In plants, pseudo-*loxP* sites were reported present in the plastid genomes [96]. Some potential ϕC31 *attB* or *attP* pseudo-sites are also identified in *Arabidopsis*[143]. Although researchers are suggesting that these pseudo-sites may be useful for providing an endogenous landing sites for site-specific integration of exogenous genes of interest [140], the presence of these pseudo-sites also pose a threat to generate aberrant chromosomes upon the expression of recombinases. The aberrant chromosomes can be generated from genomic excision, inversion or translocation through unintended recombination between these pseudo-sites. Chromosome translocation has been observed in the plant nuclear genome harboring two native *loxP* sites upon the expression of *cre* recombinase [144,145]. Unintended duplication of *loxP* or other recombination sites resulting from successive cycles of transformation (for gene stacking) and SMG elimination may cause intra- and inter-chromosomal rearrangements and lead to genome instability in the transgenic crop [146]. Also, Cre-mediated site-specific recombination, which caused unintended deletions between a native *loxP* site and a pseudo-*loxP* site in transgenic tobacco plastid genomes was observed [96]. Chromosomal re-arrangements were also observed in mouse spermatid cells (which had chronic high-level expression of a *cre* gene) and led to male sterility. One hypothesis was that the Cre-mediated genomic rearrangements occurred, perhaps at pseudo *loxP* sites within the mouse genome [147].

Next generation sequencing of crop genomes could reveal pseudo recognition sites, which would allow choosing the most appropriate recombination system by crop[148]. In addition, using a recombinase that has a very long recognition site, such as the CinH-*RS2* system (110 bp for the *RS2* site) [149] would likely have fewer off-effects from unintended DSBs in the plant genome since these recognition sites would be exceedingly rare-to-absent in most plant genomes. Again, genomic sequencing will be informative in that regard.

### Needs for future research using site-specific recombination for SMG removal

#### Explore other novel site-specific recombination systems for plant transgenesis

There are more than 100 prokaryotic site-specific recombination systems are known to exist. So far, only a few systems have been explored and utilized for plant genome manipulation. Programs with combined use of efficient multiple site-specific recombination systems for multiple functions in plant genomes are useful [150,151].

#### Develop a system for comparison of relative recombination activity among different site-specific recombination systems

The right choice of one or multiple robust site-specific recombination systems is the most critical factor to effectively facilitate genomic manipulation in plants. By far, except for the widely-used Cre-*lox*, R-*R*, FLP-*FRT* site-specific recombination systems, several other site-specific recombination systems were also reported for SMG removal *in planta* recently. They are ParA-*MRS*[152,153], ϕC31-*att*[143], Bxb1-*att*[154,155] and CinH-*RS2*[149,153]. However, the results of recombination efficiency from each of those recombination systems were collected from different labs using different methods, species, and conditions. Thus, there is no direct comparison of the recombination efficiency among these systems. Recombination sites from different site-specific recombination systems should be built as a unit into a construct and inserted into the same genomic site for direct recombination-efficiency evaluation, thus avoiding genomic position effects in the comparisons. Multi-integrase recombination sites (*FRT*-*attP*_ϕC31_-*attP*_R4_-*attP*_TP901-1_-*attP*_Bxb1_) from FLP, ϕC31, R4, TP901-1 and Bxb1 site-specific systems were built together to evaluate the relative site-specific recombination activity (in this case, integration activity was measured) were reported in mammalian cells [156]. In the study, the actual efficacy of producing transgenic cells with the corresponding integrases has been measured. However, such a study has yet to be accomplished in plants. It would be useful if a quantitative system were developed in plants to evaluate the recombination efficiency of different site-specific recombination site by site.

#### The importance of a nuclear localization signal (NLS)

Since recombinase-mediated DNA recombination reactions occur in the nucleus of plant cells, nuclear targeting of prokaryotic-originated recombinases might vary in excision efficiency. The 38-kDa Cre protein from the most efficient Cre-*lox* system used for eukaryotic genome manipulation has been assumed to readily diffuse into the nucleus owing to its small size. However, researchers have now identified nuclear targeting determinants, which resemble eukaryotic nuclear localization signals, and these have been found to affect targeting to the nucleus [157]. Cre protein is one of the few prokaryotic proteins that have been shown to carry these NLS-like determinants. Adding an NLS to the recombinases have improved the efficiency of recombinase nuclear targeting and recombination efficiency. Cre-NLS and ϕC31-NLS (a NLS fused to the recombinase), were both reported to increase the efficiency of recombination in mammalian cells [158,159].

#### The importance of ‘codon usage’ (codon optimization)

Since most of the site-specific recombination systems are from prokaryotic organisms, plant-codon optimization should increase plant expression of recombinases. In the animal kingdom, codon-optimized ϕC31 has reported to increase the recombination efficiency (site-specific integration in this case) from 40% to 69% in *Drosophila*[160]. Raymond and Soriano also reported that the mouse codon-optimized FLP and ϕC31 recombinases, FLPo and ϕC31o, improved recombination efficiency to similar levels to that of Cre, which is the most efficient site-specific recombination system to mediate DNA recombination *in vitro* and *in vivo*[161]. Most recently, a wild type FLP (FLPwt) developed for chromosomal engineering in mammalian cells, a thermostable FLP mutant (FLPe) and the mouse codon-optimized FLP (FLPo) were used for comparison of their recombination efficiency to delete a SMG in the monocot species rice and onion [151,162]. They found that the FLPe resulted in efficient SMG excision with the relative efficiency approaching 100% in the rice while the FLPwt is ineffective in excising the SMG [151]. On the other hand, the FLPo was reported to yield similar recombination efficiency to that of Cre using a transient assay in onion cells [162]. The codon-optimized version of CinH recombinase, CinHo, was successfully used to efficiently remove SMG in tobacco pollen by using pollen-specific promoters [149]. However, direct comparison of recombination ability between the wild type CinH and CinHo was not reported. Codon-optimization is worthy to be studied for improving site-specific recombination performance in plants.

#### Use of uni-directional site-specific recombination systems

In terms of reaction mode, there are two types of recombinases: *uni-directional* and *bi-directional*. Cre, FLP and R also called *bi-directional* recombinases because the recombination reactions (co-integration and excision) are fully and freely reversible. This occurs because their two recombination sites have identical sequence. For example, the product of the hybrid site derived from the recombination of the two *loxP* sites has the same sequence of the two *loxP* site and can be used as a substrate of Cre recombinase again. Therefore, this reversibility could re-integrate an SMG even though deletion reaction is kinetically favored. ϕC31 and Bxb1 are examples of *uni-directional* site-specific recombination system. Once hybrid sites are generated from an *attB* site and an *attP* site, the subsequent *attL* and *attR* sites differ and cannot serve as substrates for recombination anymore. This means once the SMG is excised, they are not able to re-insert into the genome.

#### Implement site-specific recombination approaches into ZFN technology

As described earlier, the SMG in a cassette can be eliminated from the genomes either by meganuclease-, by site-specific recombination system-, or by ZFN-mediated approaches. However, those SMG and GOI cassettes were all randomly pre-integrated beforehand, and because the insertion of GOI in the genome is random, subsequent screening for suitable GOI-expressing lines is needed because of position effects. In contrast, targeted insertion into an endogenous genomic locus of interest is attractive for genome manipulation, including allele mutation, allele replacement and gene stacking. Transgene integration into the same chromosome location can produce alleles that express at a predictable level as well [163]. Targeted genomic insertion also improves the qualitative and quantitative functional comparison of similar transgenes [164].

**Figure 8:**
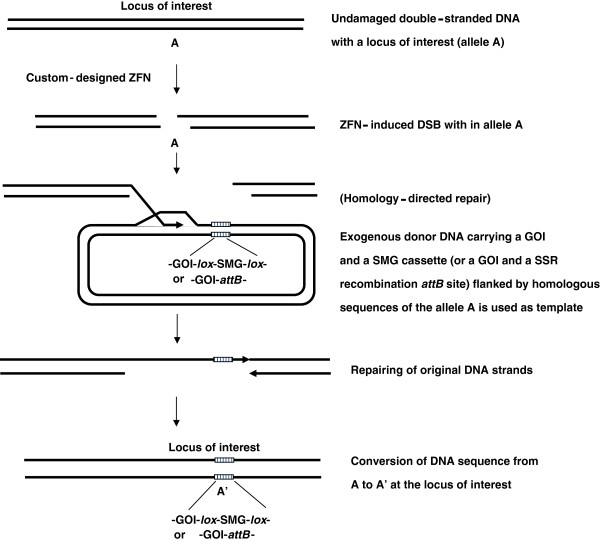
**Usage of ZFN technology to bring the site-specific recombination tool to a locus of interest for future use.** As described in Figure 7, a DSB can be induced at an allele in a locus of interest by a custom-designed ZFN and repaired through HR pathway. This in turn can introduce a GOI-*lox*-SMG-*lox* or GOI-*attB* construct, for example, into the locus. The SMG can be deleted through recombinase-mediated site-specific deletion. In the second case, the *attB* site can be used for transgene-stacking at this locus of interest. Abbreviations. GOI: gene of interest, SMG: selectable marker gene, *lox*: *loxP* site of Cre-*lox* site-specific recombination system.

As mentioned earlier, one of the most unique characteristics of ZFN technology is that a DSB at a specific locus can be induced through ZFN-mediated cleavage *in vivo* and mutate the allele by NHEJ or replace the original allele with researcher-designed donor DNA sequence by homologous recombination (HR) repairing machinery. Therefore, GOI, SMG and site-specific recombination system components can be built on a same plasmid and inserted into the locus of interest by custom-designed ZFN initially, then site-specific recombination system can be used later to remove the unwanted DNA fragment (*ex.* SMG or integrated gene) or adding genes (gene stacking) (Figure 8). The GOI could be a functional allele used to replace an endogenous defective allele or a gene used to disrupt a functional allele [122,123]. Through ZFN-induced homologous recombination, the GOI-*lox*-SMG-*lox* can be integrated into the locus of interest. The SMG assists researchers to select the integration cell lines and can be removed later by a site-specific recombination system. Using an SMG gene, such as *puro*^*R*^ or *hyg*^*R*^, to assist the screening of transgenic clones generated by ZFN-mediated gene targeting has been reported in human cells [165,166]. Alternatively, a site-specific recombination site, such as a ϕC31 *attP* site, can be brought into the locus of interest and later used for gene stacking through *attP* × *attB* sites recombination. In this case, a plasmid contains a ϕC31 *attB* site and a chosen GOI for stacking need to be provided [167]. Site-specific recombination systems are user-friendly molecular tools for eukaryotic cell genome editing without the complicated design and testing of ZFN arrays in ZFN technology. An approach of combining ZFNs for initial targeting of the site-specific recombination system to a locus of interest and subsequent use of site-specific recombination system for genome editing and SMG removal should be welcomed by many researchers.

#### The possible use of TALENs for marker gene removal

**Figure 9:**
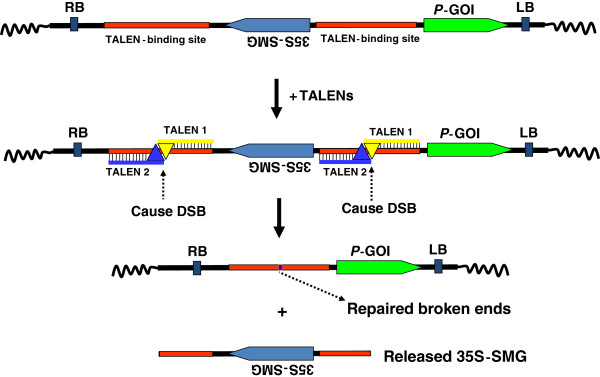
**Strategy for SMG removal in transgenic plants with TALENs.** A T-DNA containing GOI and 35S-SMG (flanked by two engineered identical TALEN-binding sites) are delivered into plant genome. Upon the expression of TALEN genes, TALEN nucleases bind to the TALEN-binding sites and induce DSBs, resulting in the release of the SMG fragment. The broken ends are repaired through endogenous repairing system. Abbreviations. RB: T-DNA right border, LB: T-DNA left border, GOI: gene of interest, SMG: selectable marker gene, P: promoter.

Transcription activation–like effector (TALE) proteins are produced by the plant pathogen *Xanthomonas*, and TALE family members are targeted to key plant genes [168]. In plants, TALEs localize to the nucleus and bind to target promoter region to trigger the expression of plant genes [169]. Recently, by taking advantage of their specific DNA binding properties, researchers fused the restriction domain of FokI nuclease to these TALEs to create TALE-FokI nuclease (TALEN) hybrid proteins [170] and use them for biotechnological application such as gene targeting (reviewed by Scholze and Boch [171]). TALENs have used to successfully knockout genes in fish and mammals (rats and human) [172-174]. TALENs have been used to knockout target genes in plants, such as the *ADH1* gene in *Arabidopsis thaliana* protoplasts [174], and used to produce disease-resistant rice [175]. Similar to ZFN technology, TALENs cause DNA double strand breakage (DSB) at a specific locus. As the result, the broken DNA is repaired through homologous recombination (HR) or NHEJ repairing pathways. Two identical sets of TALEN-binding sequences can be designed to flank a SMG in a transformation vector. After expression of TALEN, DSB will be induced at both TALEN-binding sequences and release the SMG (Figure 9). This strategy can be devised for maker-free transgenic plant production, or native chromosome fragment removal.

## Conclusions and perspectives

Genetic transformation is an important technology for biology fundamental research and for engineering transgenic organisms, including plants. SMGs have been very useful to enable plant transformation, yet there are a number of regulatory concerns of retaining SMGs in commercialized transgenic plants, leading us to conclude that ideally, the SMG should be removed after transformation. The regulatory concerns seem to focus on horizontal gene transfer of bacterially-derived SMGs from transgenic plants to bacteria. This could be obviated with SMGs derived from plants such as the ABC transporter from *Arabidopsis* that confers kanamycin resistance [176]. It is a very large gene with plant-codon-optimization that would likely not be horizontally transferred to bacteria, and even if it would be, it has proven to be not very effective as a resistance gene in bacteria [177], and thus, would likely not be selected. Innovation for SMG removal will continue, and we shall, no doubt, see improvements in existing systems and new technologies such as TALENs be configured for this purpose. Of particular importance is precision and robustness of removal without unintended consequences. Certainly, using the least amount of DNA possible is important for intellectual property and government regulatory concerns. Efficient systems that can be deployed in a multitude of crop systems should have the most value.

## Abbreviations

SMG: Selectable marker gene; GOI: Gene of interest; LoxP: Locus of crossing-over P1; Cre: Causes recombination recombinase; HR: Homologous recombination; RB: Right border of T-DNA; LB: Left border of T-DNA; ICR: Intra-chromosomal recombination; DH: Double haploid; TIR: Terminal inverted repeat; PVX: Potato virus X; ZFN: Zinc finger nuclease; GR: Glucocorticoid receptor; NHEJ: Non homologous end joining; ptDNA: Plastid DNA; hFIX: Human, Factor IX; attB: Bacterial attachment site; attP: Phage attachment site; NLS: Nuclear localization signal; codA: *Cytosine deaminase* gene; 5-FC: 5-fluorocytosine; 5-FU: 5-fluorouraci; iaaH: Indole acetamide hydrolase gene; NAM: Naphthalene acetamide; NAA: Naphthaleneacetic acid; PPT: Phophinothricin; MYMV: Mungbean yellow mosaic virus; NPTII: Neomycin phosphotransferase; PCR: Polymerase chain reaction; Bar: Bialaphos resistance; T-DNA: Transfer DNA; TrAP: Transcriptional activator protein gene.

## Competing interests

The authors declare that they have no competing interests.

## Authors’ contributions

YYY drafted the original manuscript and YYY and CNS substantially contributed to the content of the paper. All authors read and approved the final manuscript.
